# Supplementary material for: Design and Optimization of Orally Administered Luteolin Nanoethosomes to Enhance Its Anti-Tumor Activity against Hepatocellular Carcinoma
Source: Pharmaceutics. 2021 May 2;13(5):648. doi: 10.3390/pharmaceutics13050648 (PMC8147467; doi:10.3390/pharmaceutics13050648)
Supplement: Supplementary file 1 [file pharmaceutics-13-00648-s001.zip › pharmaceutics-1199433-supplementary.pdf]

pharmaceutics

MDPI

Supplementary Materials: Design and Optimization of Orally Administered Luteolin Nanoethosomes to Enhance Its Anti-Tumor Activity against Hepatocellular Carcinoma

Mahmoud M. A. Elsayed, Tarek M. Okda, Gamal M. K. Atwa, Gamal A. Omran, Atef E. Abd Elbaky and Abd El hakim Ramadan

Table S1. The accuracy of the used spectrophotometric procedure.

Scheme	LUT		
	Taken (μg \mL)	Found (μg \mL)	% recovery [a]
**1**	2	2.002	100.1
**2**	4	3.98	99.5
**3**	8	7.97	99.5
**4**	12	11.916	99.3
**5**	16	15.84	99
**Mean**		99.48	
**SD**		0.40	
**RSD**		0.40	
**RE**		0.52	

SD: Standard deviation, RSD: Relative Standard deviation; RE: Relative error; a: mean of three replicate measurements.

Table S2. The intraday and interday precision profile.

Precision level	LUT			
	Conc. (μg \mL))	% Recovery [a]	± SD	RSD
**Intraday**	2	99.9	± 0.21	0.21
	8	99.7	± 0.12	0.12
	16	99.9	± 0.98	0.98
**Interday**	2	100	± 0.83	0.83
	8	99.5	± 0.21	0.21
	16	100.5	± 1.24	1.24

SD: Standard deviation; RSD: relative standard deviation; a: Mean of three replicate measurements.

Table S3. The calculated correlation coefficient for the in vitro release of LUT from the prepared ethosomes.

Formula No.	The Correlation Coefficient (r)				
	Zero	First	Second	Diffusion	Best Fitted Order
F1	0.746577	0.799826	0.844784	0.859448	Diffusion
F2	0.716695	0.778696	0.826013	0.83558	Diffusion
F3	0.714786	0.780787	0.831069	0.834169	Diffusion
F4	0.779614	0.815357	0.844089	0.884511	Diffusion
F5	0.799451	0.845187	0.876062	0.89952	Diffusion
F6	0.806757	0.854415	0.884397	0.904771	Diffusion
F7	0.753357	0.78001	0.804955	0.861196	Diffusion
F8	0.793695	0.830608	0.859167	0.895067	Diffusion
F9	0.772838	0.824903	0.86315	0.881514	Diffusion

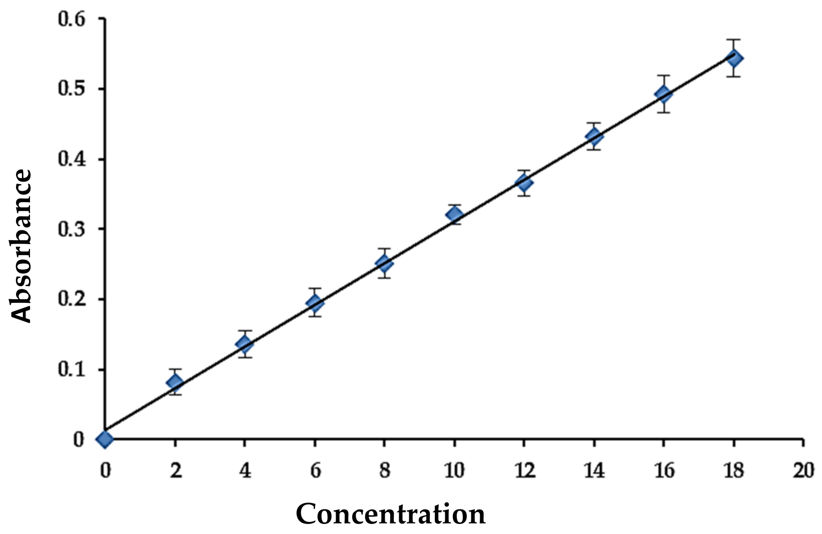

Figure S1. Calibration curve of LUT in PBS, pH 7.4 (Mean ± SD, n = 3).

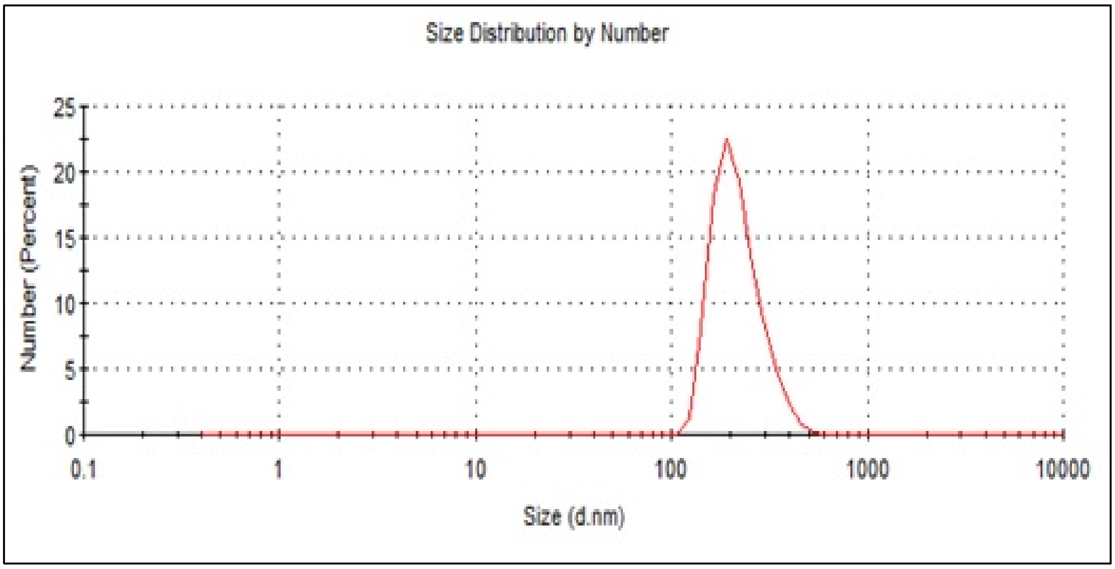

Figure S2. Vesicle size of the selected LUT loaded ethosomal formulation (F8).

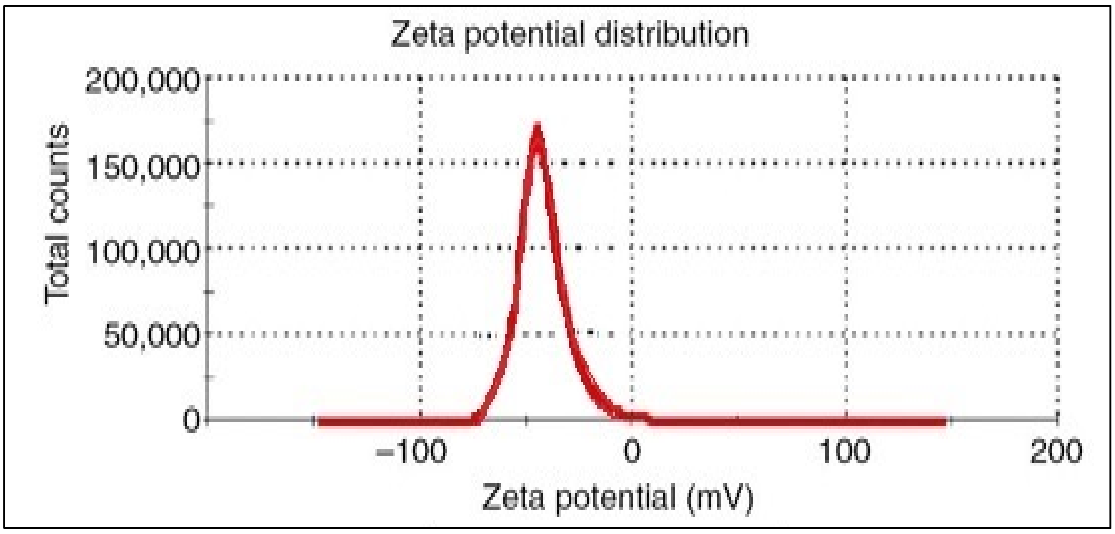

Figure S3. Zeta potential of the selected ethosomal formulation (F8).
